# Supplementary material for: Time-series transcriptome analysis identified differentially expressed genes in broiler chicken infected with mixed Eimeria species
Source: Front Genet. 2022 Aug 8;13:886781. doi: 10.3389/fgene.2022.886781 (PMC9393255; doi:10.3389/fgene.2022.886781)
Supplement: Supplementary file 2 [file DataSheet1.ZIP › 4dpi_KEGG.Gsea.1625100298584/KEGG_TIGHT_JUNCTION.html]

Details for gene set KEGG\_TIGHT\_JUNCTION[GSEA]

|  || Dataset | TMM\_4dpi\_gct\_format\_4dpi\_gct\_format.Class\_4dpi.cls #PC\_versus\_NC.Class\_4dpi.cls #PC\_versus\_NC\_repos |
| Phenotype | Class\_4dpi.cls#PC\_versus\_NC\_repos |
| Upregulated in class | 1 |
| GeneSet | KEGG\_TIGHT\_JUNCTION |
| Enrichment Score (ES) | 0.41302967 |
| Normalized Enrichment Score (NES) | 1.5834213 |
| Nominal p-value | 0.0042386698 |
| FDR q-value | 0.26342946 |
| FWER p-Value | 0.835 |
Table: GSEA Results Summary

  

Fig 1: Enrichment plot: KEGG\_TIGHT\_JUNCTION      
 Profile of the Running ES Score & Positions of GeneSet Members on the Rank Ordered List

  

| SYMBOL | TITLE | RANK IN GENE LIST | RANK METRIC SCORE | RUNNING ES | CORE ENRICHMENT || 1 | IGSF5 | na | 238 | 1.282 | 0.0213 | Yes |
| 2 | RAB3B | na | 274 | 1.207 | 0.0573 | Yes |
| 3 | CGN | na | 445 | 1.020 | 0.0759 | Yes |
| 4 | PRKCD | na | 605 | 0.875 | 0.0907 | Yes |
| 5 | TJP2 | na | 629 | 0.860 | 0.1165 | Yes |
| 6 | ACTN4 | na | 673 | 0.838 | 0.1399 | Yes |
| 7 | MYH9 | na | 704 | 0.817 | 0.1637 | Yes |
| 8 | CLDN7 | na | 832 | 0.746 | 0.1770 | Yes |
| 9 | PRKCA | na | 853 | 0.736 | 0.1991 | Yes |
| 10 | PPP2R2C | na | 970 | 0.684 | 0.2114 | Yes |
| 11 | PARD6B | na | 973 | 0.684 | 0.2332 | Yes |
| 12 | ACTB | na | 1026 | 0.663 | 0.2502 | Yes |
| 13 | SPTAN1 | na | 1047 | 0.655 | 0.2696 | Yes |
| 14 | PRKCI | na | 1053 | 0.654 | 0.2903 | Yes |
| 15 | KRAS | na | 1397 | 0.548 | 0.2791 | Yes |
| 16 | LLGL2 | na | 1429 | 0.538 | 0.2939 | Yes |
| 17 | TJP3 | na | 1445 | 0.534 | 0.3098 | Yes |
| 18 | CTTN | na | 1458 | 0.531 | 0.3259 | Yes |
| 19 | YES1 | na | 1460 | 0.530 | 0.3429 | Yes |
| 20 | PRKCE | na | 1693 | 0.476 | 0.3387 | Yes |
| 21 | AFDN | na | 1708 | 0.473 | 0.3528 | Yes |
| 22 | CTNNA2 | na | 1774 | 0.461 | 0.3622 | Yes |
| 23 | AMOTL1 | na | 1961 | 0.429 | 0.3604 | Yes |
| 24 | TJAP1 | na | 1993 | 0.423 | 0.3715 | Yes |
| 25 | CTNNA1 | na | 2131 | 0.400 | 0.3728 | Yes |
| 26 | MYH15 | na | 2178 | 0.392 | 0.3816 | Yes |
| 27 | RHOA | na | 2375 | 0.366 | 0.3769 | Yes |
| 28 | MYL12A | na | 2398 | 0.362 | 0.3867 | Yes |
| 29 | AKT3 | na | 2430 | 0.357 | 0.3956 | Yes |
| 30 | EPB41L3 | na | 2549 | 0.341 | 0.3967 | Yes |
| 31 | EXOC3 | na | 2583 | 0.337 | 0.4048 | Yes |
| 32 | CASK | na | 2614 | 0.334 | 0.4130 | Yes |
| 33 | PPP2R2B | na | 2769 | 0.312 | 0.4102 | No |
| 34 | PATJ | na | 2948 | 0.290 | 0.4046 | No |
| 35 | GNAI1 | na | 3115 | 0.267 | 0.3992 | No |
| 36 | EPB41L1 | na | 3311 | 0.242 | 0.3907 | No |
| 37 | PPP2R2A | na | 3550 | 0.213 | 0.3776 | No |
| 38 | PARD3 | na | 3629 | 0.205 | 0.3776 | No |
| 39 | ACTN1 | na | 3764 | 0.191 | 0.3725 | No |
| 40 | CTNNB1 | na | 3857 | 0.182 | 0.3707 | No |
| 41 | MPDZ | na | 3905 | 0.176 | 0.3724 | No |
| 42 | CLDN11 | na | 3926 | 0.175 | 0.3763 | No |
| 43 | MAGI2 | na | 3950 | 0.172 | 0.3800 | No |
| 44 | CLDN23 | na | 4021 | 0.166 | 0.3794 | No |
| 45 | PPP2R2D | na | 4065 | 0.162 | 0.3810 | No |
| 46 | PPP2CA | na | 4110 | 0.159 | 0.3824 | No |
| 47 | SRC | na | 4500 | 0.124 | 0.3538 | No |
| 48 | MAGI1 | na | 4789 | 0.098 | 0.3328 | No |
| 49 | CLDN2 | na | 5140 | 0.066 | 0.3056 | No |
| 50 | MAGI3 | na | 5157 | 0.064 | 0.3063 | No |
| 51 | CDC42 | na | 5190 | 0.061 | 0.3056 | No |
| 52 | CLDN1 | na | 5284 | 0.055 | 0.2996 | No |
| 53 | AKT1 | na | 5330 | 0.052 | 0.2975 | No |
| 54 | MAP3K20 | na | 5415 | 0.045 | 0.2919 | No |
| 55 | MPP5 | na | 5418 | 0.044 | 0.2931 | No |
| 56 | MYH10 | na | 5575 | 0.029 | 0.2810 | No |
| 57 | CLDN5 | na | 5865 | 0.004 | 0.2569 | No |
| 58 | CSNK2B | na | 5872 | 0.004 | 0.2565 | No |
| 59 | ASH1L | na | 5945 | -0.002 | 0.2505 | No |
| 60 | PRKCZ | na | 5974 | -0.004 | 0.2483 | No |
| 61 | LLGL1 | na | 6062 | -0.012 | 0.2414 | No |
| 62 | TJP1 | na | 6261 | -0.027 | 0.2257 | No |
| 63 | PRKCH | na | 6364 | -0.035 | 0.2182 | No |
| 64 | OCLN | na | 6770 | -0.068 | 0.1864 | No |
| 65 | RRAS | na | 6935 | -0.083 | 0.1754 | No |
| 66 | PARD6G | na | 7046 | -0.093 | 0.1691 | No |
| 67 | CSNK2A1 | na | 7174 | -0.106 | 0.1619 | No |
| 68 | PTEN | na | 7367 | -0.123 | 0.1498 | No |
| 69 | PPP2CB | na | 7527 | -0.137 | 0.1408 | No |
| 70 | NRAS | na | 7621 | -0.146 | 0.1377 | No |
| 71 | YBX3 | na | 7689 | -0.152 | 0.1370 | No |
| 72 | MRAS | na | 7800 | -0.161 | 0.1330 | No |
| 73 | MYL9 | na | 7870 | -0.168 | 0.1326 | No |
| 74 | CSNK2A2 | na | 8097 | -0.188 | 0.1197 | No |
| 75 | GNAI2 | na | 8321 | -0.211 | 0.1078 | No |
| 76 | MYL12B | na | 8519 | -0.232 | 0.0987 | No |
| 77 | JAM3 | na | 8852 | -0.268 | 0.0795 | No |
| 78 | CLDN3 | na | 8863 | -0.269 | 0.0873 | No |
| 79 | EXOC4 | na | 8948 | -0.279 | 0.0893 | No |
| 80 | EPB41 | na | 8952 | -0.279 | 0.0980 | No |
| 81 | GNAI3 | na | 9238 | -0.314 | 0.0842 | No |
| 82 | HRAS | na | 9244 | -0.315 | 0.0939 | No |
| 83 | VAPA | na | 9496 | -0.347 | 0.0841 | No |
| 84 | HCLS1 | na | 9860 | -0.394 | 0.0663 | No |
| 85 | MYH11 | na | 9968 | -0.409 | 0.0705 | No |
| 86 | JAM2 | na | 10362 | -0.472 | 0.0527 | No |
| 87 | PRKCB | na | 10600 | -0.519 | 0.0495 | No |
| 88 | CLDN10 | na | 11621 | -0.890 | -0.0074 | No |
| 89 | CTNNA3 | na | 11880 | -1.240 | 0.0109 | No |
Table: GSEA details [plain text format]

  

Fig 2: KEGG\_TIGHT\_JUNCTION      
 Blue-Pink O' Gram in the Space of the Analyzed GeneSet

  

Fig 3: KEGG\_TIGHT\_JUNCTION: Random ES distribution      
 Gene set null distribution of ES for **KEGG\_TIGHT\_JUNCTION**
